# Chronic inhibition of GABA synthesis in the infralimbic cortex facilitates conditioned safety memory and reduces contextual fear

**DOI:** 10.1038/s41398-020-0788-8

**Published:** 2020-04-24

**Authors:** Judith C. Kreutzmann, Markus Fendt

**Affiliations:** 1 Institute for Pharmacology & Toxicology, Otto-von-Guericke University Magdeburg, Magdeburg, Germany; 2 Leibniz Institute for Neurobiology, Magdeburg, Germany; 3 Center of Behavioral Brain Sciences, Otto-von-Guericke University Magdeburg, Magdeburg, Germany

**Keywords:** Learning and memory, Physiology

## Abstract

Accurate discrimination between danger and safety cues is essential for survival. Recent findings in humans indicate that patients suffering from anxiety disorders cannot reliably use safety cues in order to inhibit fear responses. However, the neuroanatomical pathways of conditioned safety are still unclear. Aim of the present study was to investigate whether chronic inhibition of GABA synthesis in the infralimbic (IL) cortex, a critical region for fear inhibition, would lead to enhanced conditioned safety memory. Male Sprague Dawley rats were equipped with osmotic mini-pumps attached to an infusion cannula aimed at the IL. Mini-pumps were either filled with the glutamate decarboxylase (GAD) inhibitor l-allylglycine (l-AG) or the inactive enantiomer d-allylglycine (d-AG). Previous studies demonstrated that chronic infusions of l-AG lead to lower GABA levels and overall enhanced neural activity. The effect of IL disinhibition on conditioned safety was investigated utilizing the acoustic startle response. Chronic disinhibition of the IL facilitated conditioned safety memory, along with reduced contextual fear and lower corticosterone levels. The present findings suggest that the IL is a key brain region for conditioned safety memory. Because anxiety disorder patients are often not capable to use safety cues to inhibit unnecessary fear responses, the present findings are of clinical relevance and could potentially contribute to therapy optimization.

## Introduction

The acoustic startle response (ASR) is a reflexive physiological reaction to an intense and sudden noise that can be observed across all mammals^1^. The ASR is modulated by a number of factors, including the individual’s arousal and affective state^1–4^. In rodents, ASR can be modified in response to emotional situations, with the startle magnitude being increased by fearful/threatening stimuli (also referred to as fear-potentiated startle (FPS))^3,5–8^ and attenuated by threat-reducing or joyful/rewarding stimuli^3,7,9–11^. Because these ASR modulations can be observed in both humans and rodents, the ASR provides an excellent translational research tool to investigate symptoms characterizing neuropsychiatric disorders^6,7,12,13^.

As such, several correlates of modulated ASR have been described in patients suffering from different neuropsychiatric disorders, including schizophrenia, Huntington’s disease, Tourette’s syndrome or anxiety disorders^14–21^. For instance, patients suffering from anxiety-related disorders, such as obsessive-compulsive disorder or post-traumatic stress disorder (PTSD), display an increased overall startle magnitude and enhanced FPS^22,23^. Moreover, impaired fear inhibition, as measured by startle attenuation, could be observed during fear extinction or conditioned safety learning^20,21,24–26^. Hence, impairments in conditioned safety have repeatedly been discussed as a biomarker of anxiety disorders^25,27–29^.

Conditioned safety is a type of associative learning process in which a safety signal indicates the non-occurrence of an aversive incident, thereby inhibiting fear and stress responses^30,31^. Due to the biological importance of conditioned safety, several studies in rodents have tried to investigate the neuronal mechanisms underlying this type of learning. Nevertheless, lesion or inactivation studies investigating the necessity of specific brain regions known to be important for fear or fear inhibition have often failed to report definitive answers regarding the involvement of the central amygdala^32^, the ventromedial prefrontal cortex (vmPFC)^33^, the auditory thalamus^34^, the nucleus accumbens^35^ or the periaqueductal gray (PAG)^36,37^ in conditioned safety learning. Interestingly, the rat vmPFC can be subdivided into two distinct structures which have been proposed to have opposing functions: While the prelimbic cortex (PL) has been suggested to mediate fear expression, the infralimbic cortex (IL) seems to be a key orchestrator in fear inhibition^38,39^. Recently, studies using either a complex discriminative protocol^40,41^ or a single-cued safety paradigm^42^ demonstrated that the IL plays a crucial role in the expression of conditioned safety. Therefore, we further wanted to characterize the role of the IL in conditioned safety.

Aim of the present study was to investigate whether chronic activation of the IL leads to enhanced conditioned safety memory. For this, we chronically infused the glutamate decarboxylase (GAD) inhibitor l-allylglycine (l-AG) into the IL using osmotic mini-pumps. Previous studies investigating the pharmacodynamics of L-AG demonstrated that l-AG-induced GAD inhibition blocks γ-aminobutyric acid (GABA) biosynthesis, which in turn leads to lower GABA levels and overall enhanced neural activity (see also Supplementary Fig. S1)^43–45^. As control substance we infused the inactive enantiomer d-allylglycine (d-AG) which does not affect GABA synthesis^44^. Since conditioned safety is measured during the exposure to a fearful context, we further evaluated the conditioned contextual fear response, as well as the associated increase of peripheral corticosterone (CORT). Utilizing the ASR paradigm also allowed us to measure the prepulse inhibition (PPI) of the ASR. PPI is the reduction of the startle magnitude when the startle stimulus is preceded by a weak (non-startling) sensory stimulus (prepulse), and is widely used as an operational measure for sensorimotor gating^6^. Since acute disinhibition of the vmPFC has been shown to impair PPI^46^, we additionally measured PPI as a positive behavioral control.

## Material and methods

### Animals and housing conditions

Experimental subjects were adult male Sprague Dawley rats (*n* = 24), aged 8 weeks. Rats were bred in our animal facility (original breeding stock: Taconic, Denmark) and weighed between 260 and 310 g. Animals were group-housed in transparent Makrolon Type IV cages (1820 cm^2^) with wood chip bedding, nesting material and cage enrichment. The animals had free access to standard chow (Ssniff^®^ R/M-H, V1534–0) and tap water, with a fixed 12:12 h light/dark photoperiod (lights on at 06:00 h) in a temperature- (22 ± 2 °C) and humidity-controlled room (50 ± 5%).

All experimental procedures were approved by the local authorities (Landesverwaltungsamt Sachsen-Anhalt, 42502-2-1309 Uni MD) and conducted in agreement with international guidelines and regulations for animal experiments (2010/63/EU).

### Pharmacological intervention

For chronic inhibition of GABA synthesis in the IL, osmotic mini-pumps (Model 1002, Alzet, Charles River Laboratories, Sulzfeld, Germany) were either filled with the GAD inhibitor l-Allylglycine (l-AG; ChemCruzsc-255236; Santa Cruz Biotechnology Inc., Heidelberg, Germany) or the inactive enantiomer d-Allylglycine (d-AG; ChemCruz sc-218013; Santa Cruz Biotechnology Inc., Heidelberg, Germany). The rats were randomly assigned to the treatment groups (50%: l-AG; 50%: d-AG). The mini-pumps were implanted subcutaneously and connected to an implanted injection cannula (see below) via a vinyl tube (Plastic One Inc., Bilaney Consultants, Düsseldorf, Germany). The mini-pumps released 7.0 nmoles/0.25 μl/h for a minimal duration of 14 days. To assure that animals received the substance, mini-pumps were weighed empty, after filling them (at the time of implantation) and at the end of the experiment.

### Cannula and mini-pump implantation

For cannula implantation in the IL, animals underwent stereotactic surgery 6–7 days prior to behavioral testing. Rats were anaesthetized with isoflurane (2.5–3.5%; Baxter, Germany), mounted onto a stereotactic apparatus with a heating pad, and the filled mini-pumps were subcutaneously implanted caudal to the left shoulder blade. Two sterilized stainless-steel anchor screws were inserted into the skull, and one single stainless-steel osmotic pump connector cannula (328OPT/SPC; Plastics One Inc., Bilaney Consultants, Düsseldorf, Germany) was stereotactically implanted into the midline between the IL (AP, +2.5; ML, ±0; DV, −5.0). The cannula was fixed to the skull with dental cement (Paladur®, Heraeus Kulzer, Hanau, Germany). Rats were removed from the stereotactic apparatus, injected subcutaneously with carprofen (*Rimadyl*; 5 mg/kg s.c.) to prevent post-surgical pain, and observed until they return to consciousness. Following 24 h of single-housed recovery, rats were returned to their home cage in groups of 4–6. Post-surgical treatment included body weight check-ups and behavioral well-being.

### Behavioral testing

**Fig. 1:**
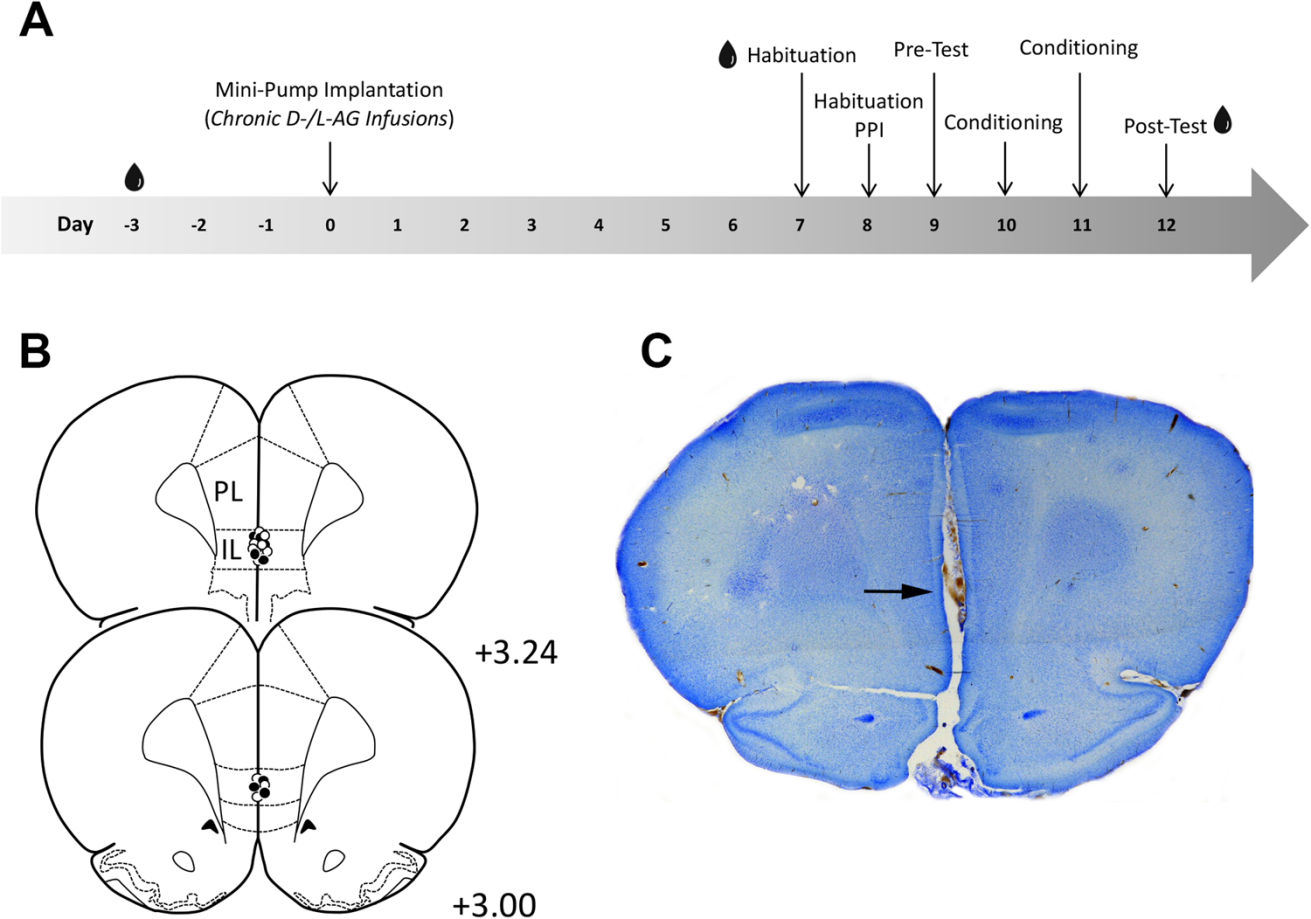
Experimental design of the study and cannula placement into the infralimbic cortex. **a** Timeline of the study: upon 2 days of handling and the first blood sample (Day −3), male Sprague Dawley rats underwent stereotactic cannulation and mini-pump implantation (d-/l-AG Infusions, Day 0). Following 6–7 days of recovery, a second blood sample (Day 7) was drawn before submitting rats to the first startle habituation session (Day 7). 24 hours later, rats underwent a second startle habituation session (Day 8), as well as the prepulse inhibition test (PPI). Rats were then submitted to our safety conditioning procedure: First, the Pre-Test was conducted (Day 9), followed by two safety conditioning sessions (Day 10 and 11). On the last day we tested for conditioned safety memory in the expression session (Post-Test, Day 12) followed by a final blood sample. **b** Infusion cannula placements in the infralimbic cortex (IL) and photomicrograph of a representative cannula tract (**c**). Open symbols in **b** represent individuals of the d-AG group, whereas filled symbols represent l-AG-treated rats; the numbers in **b** indicate the distance of the histology plate anterior to bregma.

Due to the reservoir volume of the mini-pumps, experimental procedures were conducted within 14 days, with a 5–6-day recovery period following surgery. The experimental procedures of the present experiment took place as depicted in Fig. 1a. During the experiment, the investigator was blind to the rat’s treatment.

#### Startle setup

For the measurement of PPI, conditioned safety and conditioned contextual fear a computerized startle system (SR-LAB, San Diego Instruments, USA) with eight chambers (35 cm × 35 cm × 38 cm) was used. Each chamber was equipped with a loudspeaker, a light source (10 W light bulb, ~1000 lux) and a transparent animal enclosure (9 cm × 20 cm). As startle stimuli noise, bursts with a duration of 40 ms and an intensity of 96 dB SPL were used. As aversive stimuli, scrambled electric stimuli (0.5 s, 0.6 mA) were administered via a floor grid. The delivery of the startle, light and electric stimuli was controlled by the SR-LAB software.

The responses to the startle bursts or the electric stimuli were measured by piezoelectric motion sensors underneath of the animal enclosures. The output signal of the sensor was digitalized at a sampling rate of 1 kHz, send to the computer and further analyzed by the SR-LAB software. Sequenced 1-ms readings were recorded at the stimulus onset to obtain the magnitude of the rat’s response to the startle stimulus (arbitrary units). Startle magnitude was quantified by averaging the mean sensor output in the startle response peak window 10–30 ms after startle stimulus onset.

#### Prepulse inhibition (PPI)

Following 5 min of acclimatization (only background noise), 12 startle stimuli (white noise, 40 ms, 108 dB SPL) with an inter-trial-interval (ITI) of 20 s were presented to habituate the startle response. Afterwards, six blocks were presented, each including a startle stimulus alone or startle stimuli with prepulses of 2, 4, 8, 12 or 16 dB SPL above background noise (60 dB SPL) in a pseudo-randomized order. All prepulses had a duration of 20 ms and preceded the startling stimulus by 100 ms (onset to onset).

#### Conditioned safety

Behavioral experiments were performed during the first hours of the light phase with a safety conditioning protocol described in detail elsewhere^42^. In short, on the first and second day of behavioral testing (Day 7 and 8), rats underwent startle measurements for habituation, followed by a “Pre-Test” to determine potential unconditioned effects of the to-be-learned light stimulus (Day 9): After 5 min of acclimation and 10 startle stimuli for startle baseline measurement, 20 startle stimuli were presented in a pseudo-randomized order, 10 without light (Startle Alone) and 10 upon presentation of the to-be-learned light CS (light and startle stimuli co-terminated). Rats then underwent safety conditioning for two consecutive days (Day 10 and 11): For this, rats received 15 electric stimuli (US) that were explicitly unpaired from the 5s-light CS (ITI: 12–120 s), meaning, following conditioning, the light CS would predict the absence of an aversive stimulus. On the last test day (Day 12), rats underwent a memory expression session (Post-Test) that was identical to the Pre-Test.

### Blood sampling

In order to determine corticosterone (CORT) plasma levels, blood samples were collected at three points in time: Baseline I (Day −3), Baseline II (Day 7) and after the expression session (Day 12). For blood collection, the rats were gently restrained, a small tail vain incision was made and ~40 μl of blood was collected in ethylenediamine tetraacetic acid (EDTA)-coated microtubes (Microvette® CB 300 K2E, Sarstedt AG &Co., Nümbrecht, Germany). Samples were immediately put on ice and centrifuged at 4 °C with 3000 rpm for 10 min (Eppendorf AG, Hamburg, Germany). Plasma (~15–20 μl) was collected and stored at −80 °C until further processing. Blood samples were consistently drawn in the morning and 30 min after behavioral testing between 08:30 a.m. and 10:00 a.m. Animals were handled and habituated to the blood collecting procedure prior to the first blood collection.

### Corticosterone ELISA

In order to determine CORT levels in the obtained plasma samples, an enzyme-linked immunosorbent assay (ELISA) kit specific for CORT (Enzo Life Sciences GmbH, Lörrach, Germany, Catalog No. ADI-901–097) was applied. The assay was performed as per instructions provided by the manufacturer. In short, plasma samples were diluted 1:100 in ELISA assay buffer, two 100 μl duplicates of each sample were added to the assay plate and incubated for 2 h at room temperature. After several washing steps, the substrate (p-nitrophenylphosphate, p-Npp) was added, and following one hour of incubation, the reaction was terminated and absorbance read on a microplate reader (ASYS HITECH GmbH, Eugendorf, Austria) at 405 nm.

### Histology

Animals were sacrificed, brains extracted and post-fixed in a 30% sucrose 10% formalin solution. Brains were frozen, sectioned in 50-μm thick coronal slices and directly mounted onto gelatin-coated microscope slides. Slices were Nissl-stained (5% cresyl violet) and cannula placements determined through comparison with a rat brain atlas^47^.

### Descriptive and statistical analysis

To analyze conditioned safety memory in the Post-Test, the mean startle magnitudes of the startle trials in the absence (Startle Alone) and in the presence of the light stimulus (CS-startle), as well as the absolute difference between these two means was calculated for each animal. The percent difference scores were calculated to evaluate the safety learning effect independent of potential effects on the startle alone magnitude. For the analysis of contextual fear conditioning, the baseline startle measurements, i.e. the 10 startle stimuli before the measurement of startle alone and CS startle, from the Pre- and Post-test were used. To evaluate the shock-induced activity, the mean locomotor response to the electric stimuli for analyzed. For PPI, the prepulse inhibition for each prepulse intensity was calculated for each individual animal according to the following formula: PPI = (mean startle magnitude without prepulse − mean startle magnitude with prepulse)/(mean startle magnitude without prepulse/100). Furthermore, the mean prepulse inhibition of the individual animals was calculated, i.e. the mean prepulse inhibition for all prepulse intensities.

Estimated sample size was calculated with GPower (V3.1.7) based on data of previous experiments^42^. For statistical analysis, Prism 8.0 (GraphPad Software Inc., La Jolla, CA, USA) was used. Normal distribution of the data was checked with the D’Agostino-Pearson omnibus normality test, equal variances were tested with the F-test or Bartlett’s test. Startle magnitudes and CORT data were evaluated by analyses of variance (ANOVA) with treatment as between-subject factor, and startle trial type or blood sample trial as within-subject factors. Statistical significance for percent changes in startle magnitudes and mean PPI data were analyzed with Student’s two-tailed *t*-test. Main effects and interactions were deemed significant with *p* ≤ 0.05 for all statistical tests. Between-subjects and within-subject post hoc comparisons were made using Sidak’s multiple comparisons test. A linear regression analysis was used to check whether individual conditioned contextual fear was correlated with the conditioned safety learning scores or CORT levels (see Supplementary Information, Fig. S2). Results are represented as means + SEM. Subjects with misplaced cannulas or missing startle response were excluded from analysis. Animals with misplaced injections were separately analyzed (see Supplementary Information, Fig. S3).

## Results

### Chronic l-Allylglycine infusions into the infralimbic cortex enhance the expression of conditioned safety memory

To chronically inhibit GABA synthesis in the IL, we implanted rats with infusion cannulas attached to osmotic mini-pumps that were either filled with l-AG or the inactive enantiomer d-AG. Histological analysis revealed that 11 d-AG-treated rats and 13 l-AG-treated rats had intact cannula placement (Fig. 1b). A similar cannulation procedure to bilaterally target cortical subregions with one single midline cannula has previously been used by others^48–50^.

**Fig. 2:**
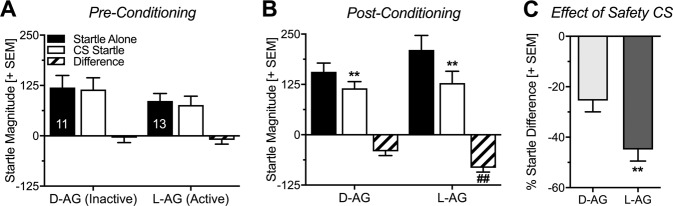
Chronic infusions of l-AG into the infralimbic cortex enhance the expression of conditioned safety. **a** Pre-conditioning, the to-be-learned safety CS had no effect on the startle response, neither in l-allylglycine (l-AG) nor in d-allylglycine (d-AG) treated rats. **b** In the expression session (Post-Conditioning), both treatment groups significantly attenuated their startle magnitude upon presentation of the safety CS (**p < 0.01, comparison with Startle Alone; ^##^*p* < 0.01, comparison with d-AG). **c** Percent difference scores confirmed that l-AG treated rats showed significantly enhanced expression of conditioned safety compared with d-AG treated rats (**p < 0.01). Data are represented as group averages + SEM. Numbers depicted in the bars represent the *n* of each group.

During the Pre-Test, both the light stimulus and the treatment (l-AG vs. d-AG) did not affect startle magnitude (Fig. 2a; ANOVA: Trial type: *F*_(1,22)_ = 0.91, *p* = 0.34; Treatment: *F*_(1,22)_ = 1.11, *p* = 0.30; interaction: *F*_(1,22)_ = 0.11, *p* = 0.73). In the expression session (Post-Test), all rats, regardless of treatment, significantly attenuated their startle magnitude in the presence of the safety CS (Fig. 2b; ANOVA: Trial type: *F*_(1,22)_ = 67.62, *p* < 0.0001), indicating that both treatment groups successfully acquired conditioned safety. This was confirmed by post hoc comparisons that showed a significant reduction of the startle response by the light CS in both treatment groups (Sidak’s multiple comparisons: d-AG: *t*_(22)_ = 3.73; *p* = 0.002; l-AG: *t*_(22)_ = 8.09, *p* < 0.0001). Notably, there was no main effect of the treatment (*F*_(1,22)_ = 0.74, *p* = 0.40) but a significant interaction between treatment and trial type (*F*_(1,22)_ = 7.47, *p* = 0.01), indicating that startle attenuation by the light CS is affected by the treatment. Post hoc comparisons further showed that startle alone (*t*_(44)_ = 1.36; *p* = 0.33) or CS startle (*t*_(44)_ = 0.33; *p* = 0.934) did not differ between d-AG and l-AG treated rats. However, both, the absolute and the percent difference scores, were significantly increased after chronic infusions of l-AG into the IL (Fig. 2b: absolute difference, *t*_(22)_ = 2.73, *p* = 0.01; Fig. 2c: percent difference, *t*_(22)_ = 3.01*, p* = 0.007), i.e. inhibition of GABA synthesis in the IL significantly enhanced the expression of safety memory. In animals with misplaced infusion cannulas, chronic l-AG infusions did not affect the expression of conditioned safety (Mann–Whitney *U*-Test: *U* = 6; *p* = 0.99; *n* = 7; Supplementary Fig. S3).

### Chronic l-Allylglycine infusions into the infralimbic cortex reduce contextual FPS and corticosterone release

**Fig. 3:**
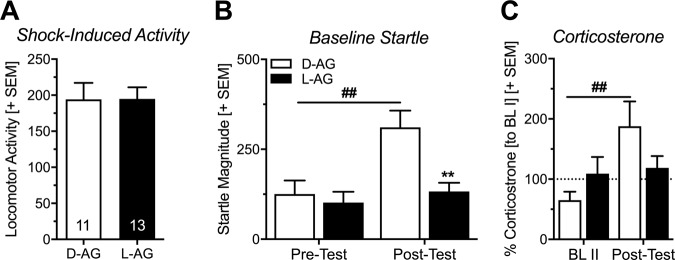
Chronic infusions of l-allylglycine into the infralimbic cortex reduce baseline startle in a fearful context and lead to lower plasma corticosterone levels. **a** Chronic inhibition of GABA synthesis in the infralimbic cortex (IL) did not affect reactivity to the aversive electric stimuli as measured by locomotor activity (arbitrary units). **b** While there was no difference between treatment groups in startle magnitude during the baseline before safety conditioning (Pre-Test), baseline startle in the expression session (Post-Test) was increased in control rats (d-Allylglycine, d-AG), suggesting contextual fear conditioning. Such increase could not be observed in L-allylglycine (l-AG) treated rats (**p < 0.01, comparison with d-AG; ^##^*p* < 0.01, comparison with Pre-Test; Sidak’s post hoc after main effects in an ANOVA). **c** Corticosterone levels were significantly enhanced in d-AG treated rats after the Post-Test, while they remained similar in l-AG treated rats (^##^*p* < 0.01, comparison with BLII; Sidak’s post hoc after main effects in an ANOVA). Data are represented as group averages + SEM. Numbers depicted in the bars represent the *n* of each group.

To check whether chronic inhibition of GABA synthesis in the IL affected the reactivity to the aversive stimuli during the safety conditioning procedure, we analyzed the locomotor response of the rats to the electric stimuli. This locomotor response was not affected by the treatment (Fig. 3a: *t*-test: *t*_(22)_ = 0.02, *p* = 0.98) which indicates that IL disinhibition does not affect the reactivity to aversive stimuli.

An increase of baseline startle magnitudes after conditioning with aversive stimuli is often used as an indicator of contextual fear conditioning^51,52^. We observed such an increase in the d-AG-treated group, while there was no increase of baseline startle in the l-AG-treated group (Fig. 3b; ANOVA: session: *F*_(1, 22_ = 10.63, *p* = 0.004; treatment: *F*_(1,22)_ = 7.94, *p* = 0.01; interaction: *F*_(1,22)_ = 5.41, *p* = 0.03; post hoc comparisons: d-AG group: *t*_(22)_ = 3.80, *p* = 0.002, l-AG group: *t*_(22)_ = 0.69, *p* = 0.75). This indicates that IL disinhibition blocks contextual fear.

Analysis of CORT levels revealed no main effect of treatment but a main effect of session, i.e. of conditioned safety learning, and an interaction between session and treatment (Fig. 3c; ANOVA: session: *F*_(1,22)_ = 5.56, *p* = 0.03; treatment: *F*_(1,22)_ = 0.23, *p* = 0.64; interaction: *F*_(1,22)_ = 4.07, *p* = 0.05). Post hoc comparisons showed no group difference before conditioning, i.e. in the second baseline blood sample (Sidak’s multiple comparisons: *t*_(44)_ = 1.15, *p* = 0.45). CORT levels of d-AG treated animals were increased after the expression session (Post-Test) when compared to the second baseline blood sample (*t*_(44)_ = 2.97; *p* = 0.01). This effect could not be observed in l-AG-treated rats (l-AG: *t*_(22)_ = 0.24, *p* = 0.96).

### Chronic infusions of l-Allylglycine in the infralimbic cortex lead to deficits in prepulse inhibition

**Fig. 4:**
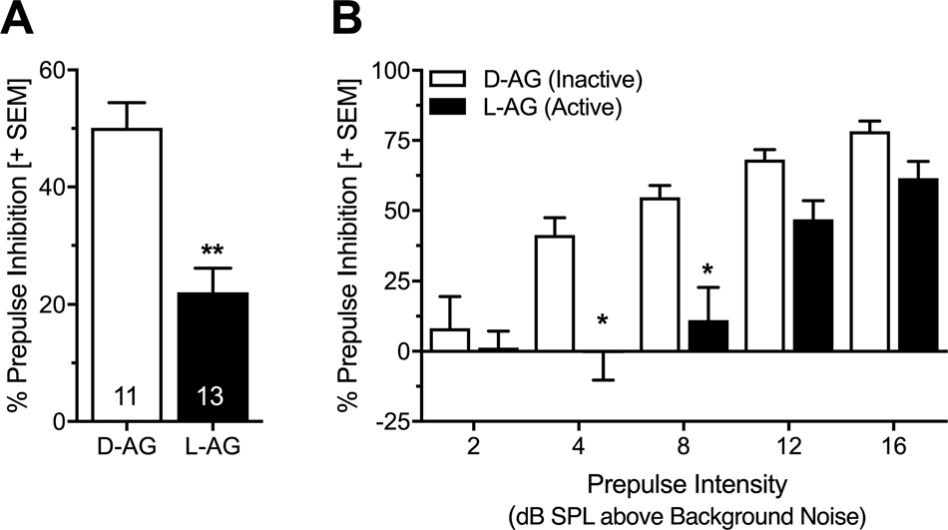
Chronic infusions of l-AG into the infralimbic cortex cause deficits in prepulse inhibition. **a** Enhanced infralimbic cortex (IL) activity generated by chronic infusions of l-allylgycine (l-AG) led to decreased mean prepulse inhibition (PPI) as compared to rats receiving the inactive enantiomer d-allylglycine (d-AG) (**p < 0.0001, Student’s *t*-test). *b* This effect was more pronounced after prepulses with low intensities (4 and 8 dB SPL) (*p < 0.001, Sidak’s post hoc after main effects in an ANOVA). Data are represented as group averages + SEM. Numbers depicted in the bars represent the *n* of each group.

Because acute activation of the vmPFC has been shown to impair PPI^46^, we further tested the effect of chronic IL disinhibition on PPI. We found that chronic IL infusions of l-AG impaired overall PPI (Fig. 4a: *t*-test: *t*_(22)_ = 4.74, *p* < 0.0001; Fig. 4b: ANOVA: treatment *F*_(1,22)_ = 17.67, *p* = 0.0004). As expected, PPI was more pronounced with higher prepulse intensities (Fig. 4b; ANOVA: prepulse Intensity: *F*_(4,88)_ = 29.13, *p* < 0.0001; interaction: *F*_(4,88)_ = 2.66, *p* = 0.04). Post hoc comparisons showed significantly reduced PPI in l-AG treated rats after prepulses with the intensity of 4 and 8 dB SPL above background noise (Sidak’s multiple comparisons: 2 dB SPL: *t*_(110)_ = 0.64, *p* = 0.97; 4 dB SPL: *t*_(110)_ = 3.89, *p* = 0.0009; 8 dB SPL: *t*_(110)_ = 4.04, *p* = 0.0005; 12 dB SPL: *t*_(110)_ = 1.97, *p* = 0.23; 16 dB SPL: *t*_(110)_ = 1.55, *p* = 0.48).

## Discussion

Aim of the present study was to investigate whether chronic disinhibition of the IL leads to enhanced expression of conditioned safety. To activate the IL, we performed chronic local infusions of the GAD inhibitor l-AG via an osmotic mini-pump system. We found that chronic inhibition of GABA synthesis in the IL facilitated the expression of conditioned safety memory, along with a reduced expression of contextual fear and reduced plasma CORT-levels. Moreover, chronic l-AG infusions into the IL caused PPI deficits.

To investigate the effect of chronic IL disinhibition on conditioned safety, we used an established protocol in which the aversive US and the safety CS were explicitly unpaired^11,31,42,53^. More specifically, both stimuli would never occur together and the safety CS thereby came to predict the absence of the aversive event. As a to-be-conditioned stimulus, we used a light cue which did not affect the ASR magnitude (Fig. 2a) in the Pre-Test, demonstrating that this light cue had no unconditioned effects on the startle response. After safety conditioning, the ASR magnitude was significantly attenuated during the presentation of the safety CS (light stimulus) in both treatment groups (Fig. 2b). Because ASR is a bivalent measure that can be potentiated by stimuli with negative and attenuated by stimuli with positive valence^13^, the present findings indicate that the associative memory to the light cue after safety conditioning had a positive valence. This is in line with previous studies of our group using the identical^42^ or similar versions of this protocol^11,54^. Importantly, the light cue does not have such startle-attenuating effects after a “pseudo-conditioning” procedure, i.e. random presentations of US and CS^42,55,56^. Thus, the type of safety conditioning (explicit unpairing) used in the present study provides a valid protocol to investigate conditioned safety. This was also shown by others using the same type of training procedure but a different species or behavioral read-out, such as freezing behavior^53,57–61^.

Analysis of the baseline startle measurements in the Pre- and Post-Tests revealed that d-AG treated control rats significantly increased their startle magnitude after safety conditioning in the expression session (Post-Test; Fig. 3b). Along with this startle potentiation by the context we observed an increase in plasma CORT levels after the expression session (Fig. 3c). Both these findings suggest that the rats associated the context with the electric stimuli, hence, they were fear conditioned to the context. Of note, this baseline startle potentiation did not correlate with the individual increase in CORT levels (Supplementary Fig. S2B). However, the individual increase in CORT level weakly correlated with the effect of the safety CS on the startle magnitude (Supplementary Fig. S2C). This indicates that rats with a higher increase in plasma CORT were not able to recall the previously learned safety memory as effectively as rats with lower plasma CORT levels.

To chronically activate the IL, we used local infusions of the GAD inhibitor l-AG via an osmotic mini-pump system. Previous studies have successfully applied this methodological approach when investigating panic- or anxiety-like behaviors, and repeatedly showed increased neural activity and reactivity after chronic l-AG infusions into the BNST or the dorsomedial hypothalamus^44,45,62,63^. Moreover, these studies were able to show that the effects observed with chronic l-AG infusions could be restored with an acute injection of the GABA_A_ receptor agonist muscimol, and that no diffusion effects of l-AG could be detected in adjacent brain areas beyond about 1 mm of radius^45,62,63^. To the best of our knowledge, there were no studies so far performing chronic l-AG infusions into the IL. However, acute infusions of l-AG into the IL increased neural activity within the IL and affected behavioral performance^64^, suggesting that also the chronic l-AG infusions used in the present study increased IL activity and reactivity (see also Supplementary Fig. S1).

Conditioned safety can be regarded as a type of fear inhibition learning that is distinct from extinction learning^31^. So far, the neuroanatomical substrates underlying conditioned safety are poorly understood. Until recently, most lesion or inactivation studies were unable to demonstrate a causal involvement of a specific brain region for conditioned safety^32–37,65,66^. Importantly, most of these studies used a conditioned inhibition protocol to induce safety learning. By using a protocol with backward pairings, lesions of the sensory insula affected safety learning^67^. However, backward pairing conditioning has also been described as “relief learning” and previous studies demonstrated that different brain areas are involved in this type of learning as compared to safety learning by an explicitly unpaired protocol^11,54^. A crucial role of the IL has been shown in different studies, either using a complex discriminative protocol^40,41^ or a single-cue paradigm, i.e. without a threat-predicting signal^42^. Based on these findings, the aim of the present study was to investigate the effects of chronic IL activation on conditioned safety memory.

We found that chronic l-AG infusions facilitated the expression of conditioned safety memory, as demonstrated by significantly stronger attenuation of startle magnitude in the presence of the safety CS (Fig. 2b, c). In addition, chronic inhibition of GABA synthesis via l-AG infusions blocked the potentiation of baseline startle by contextual fear (Fig. 3b), as well as the increase of plasma CORT (Fig. 3c). Importantly, linear regression analyses revealed that less contextual fear was not correlated with enhanced safety learning in the d-AG treated control rats and there was only a very weak correlation in the l-AG treated rats (Supplementary Fig. S2A). This indicates that disinhibition of the IL affected conditioned safety memory and contextual fear memory independently from each other.

We further found that chronic activation of the IL did not affect the reactivity to electric stimuli, as measured by locomotor response to these (Fig. 3a). This finding suggests that the observed effects on the expression of conditioned safety and contextual fear were not due to enhanced or reduced processing of the aversive event (electric stimuli). This is in line with our previous findings showing that also acute IL inhibition does not affect the reactivity to aversive stimuli^42^. Chronic disinhibition of IL neurons probably led to enhanced IL reactivity and/or to chronic inhibition of the amygdala. The basolateral amygdala (BLA) and medial PFC have been proposed to be key orchestrators of a brain circuitry responsible for the inhibition of fear^68^. The IL projects directly to a cluster of inhibitory interneurons, named intercalated cells (ITC)^68,69^, and the ITC in turn project to the BLA. Increased IL activity due to chronic l-AG infusions may therefore induce meta-plasticity in the downstream ITC and BLA neurons^70–72^, which then, in turn, inhibit the output of the central amygdala and facilitate the fear-inhibiting effects of a safety stimulus^73,74^. Furthermore, both of these processes would be able to modulate contextual fear (Fig. 3b) and plasma CORT levels (Fig. 3c). Importantly, since animals with misplaced cannulas (including the neighboring PL) did not show facilitated conditioned safety memory, these effects seem to be specific to the IL (Supplementary Fig. S3).

Our findings are in line with studies investigating fear extinction. Here, a single acute intra-IL picrotoxin injection has been shown to reduce freezing in the conditioning context and to facilitate fear extinction to the context throughout the following days, suggesting that the IL was primed by the single injection which then, in turn, led to facilitated fear extinction^75^. Although this data suggests that acute GABA inhibition may also enhance safety learning, our experiment only allows us to draw conclusions on chronic IL disinhibition. Therefore, it remains to be explored whether acute IL activation would also facilitate conditioned safety memory and whether the mechanism by which this is achieved are similar to those of chronic activation. We propose that, as part of a larger network that inhibits fear expression, the IL elicits top–down control in order to inhibit the expression of conditioned fear following conditioned safety learning. However, it would be interesting to investigate whether the chronic IL activation causes a reorganization of the fear circuitry and whether this also holds true for acute activation. Furthermore, the findings of the present study allow us to exclusively draw conclusions about the expression of conditioned safety memory. As IL inactivation before safety conditioning does not impair the acquisition of conditioned safety^42^, training-induced plasticity probably occurs in other brain structures. Therefore, our chronic IL activation, as achieved by inhibition of GABA synthesis, most probably did not enhance safety learning per se but rather increased the expression of safety memory.

In the present study we also measured the effect of chronic IL disinhibition on PPI and found a PPI deficit in animals with chronic l-AG infusions (Fig. 4). PPI is the reduction of the startle magnitude when the startle stimulus is preceded by a weak (non-startling) sensory stimulus (prepulse), and is widely used as an operational measure for sensorimotor gating^6^. The measurement of PPI was motivated by published findings that showed that acute medial PFC activation by picrotoxin injections caused PPI deficits^46^, and had the purpose to serve as a “positive behavioral control”. The PPI deficit found in the present study confirm Japha and Koch’s findings^46^, and further support an important role of the medial PFC in the modulation of PPI. Notably, the neural circuits for PPI modulation are different from those of fear expression and fear inhibition and are excellently reviewed elsewhere^76–78^.

Taken together, in the present study we chronically infused the GAD inhibitor l-AG or its inactive enantiomer d-AG into the IL. We found that l-AG treated rats displayed facilitated conditioned safety memory, along with reduced baseline startle to the context and reduced CORT level increases. We previously showed that acute IL inactivation blocked the expression of conditioned safety but had no effect on acquisition^42^. This suggests that the facilitated conditioned safety memory in the present study is probably driven by increased memory expression and not acquisition. At the first glance, this may indicate that IL stimulation during acquisition of learned safety may not be an optimal therapeutic solution for humans suffering from anxiety disorders. However, behavioral cognitive therapy usually consists of multiple learning sessions, which are simultaneously also expression sessions. IL stimulation during these sessions may facilitate expression and re-consolidation processes and, thus, lead to long-lasting conditioned safety memory and inhibition of fear. We suggest that targeted IL stimulation during behavioral cognitive therapy may facilitate therapy outcome. Non-invasive brain stimulation can be achieved by, for example, transcranial magnetic stimulation (TMS). And indeed, vmPFC stimulation by TMS has been shown to enhance fear extinction memory in healthy humans^79^. Moreover, a recent meta-analysis investigated the effect of dorsolateral PFC TMS in patients suffering from anxiety disorders, with the general finding that TMS seems to have an overall positive therapeutic effect and can be well tolerated by patients^80^. Together, these data show that TMS of the vmPFC, but specifically the IL, may be of therapeutic advantage as complementation to conventional cognitive behavioral therapy.

## Supplementary information

Supplementary Material
