# Supplementary material for: Chronic inhibition of GABA synthesis in the infralimbic cortex facilitates conditioned safety memory and reduces contextual fear
Source: Transl Psychiatry. 2020 Apr 24;10:120. doi: 10.1038/s41398-020-0788-8 (PMC7182568; doi:10.1038/s41398-020-0788-8)
Supplement: Supplementary file 1 — Supplementary Material [file 41398_2020_788_MOESM1_ESM.docx]

**Supplementary Information**

**Supplementary Figure S1**

**
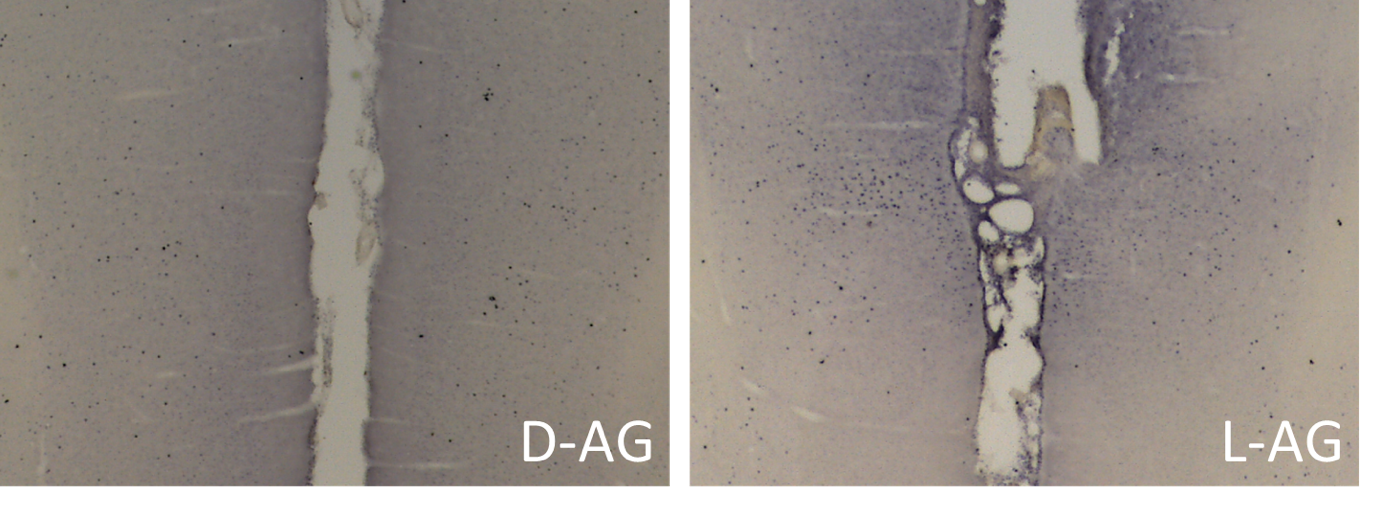
**

**Supplementary Figure S1**: **Neuronal activation in the infralimbic cortex following chronic D- or L-AG infusions.**

In a pilot experiment we investigated the effect of chronic D- and L-AG infusions on a marker of neuronal activity (cFos). In short, animals were implanted with osmotic mini-pumps which released D- or L-AG for 12 days. On Day 13, animals were perfused, the brains fixated and immunohistochemistry for cFos performed (primary antibody: cFos (E-8): sc-166940, Santa Cruz Biotechnology). Immunohistochemical representations showed higher cFos expression in L-AG treated rats (right) as compared to D-AG treated rats (left).

**Supplementary Figure S2**

**
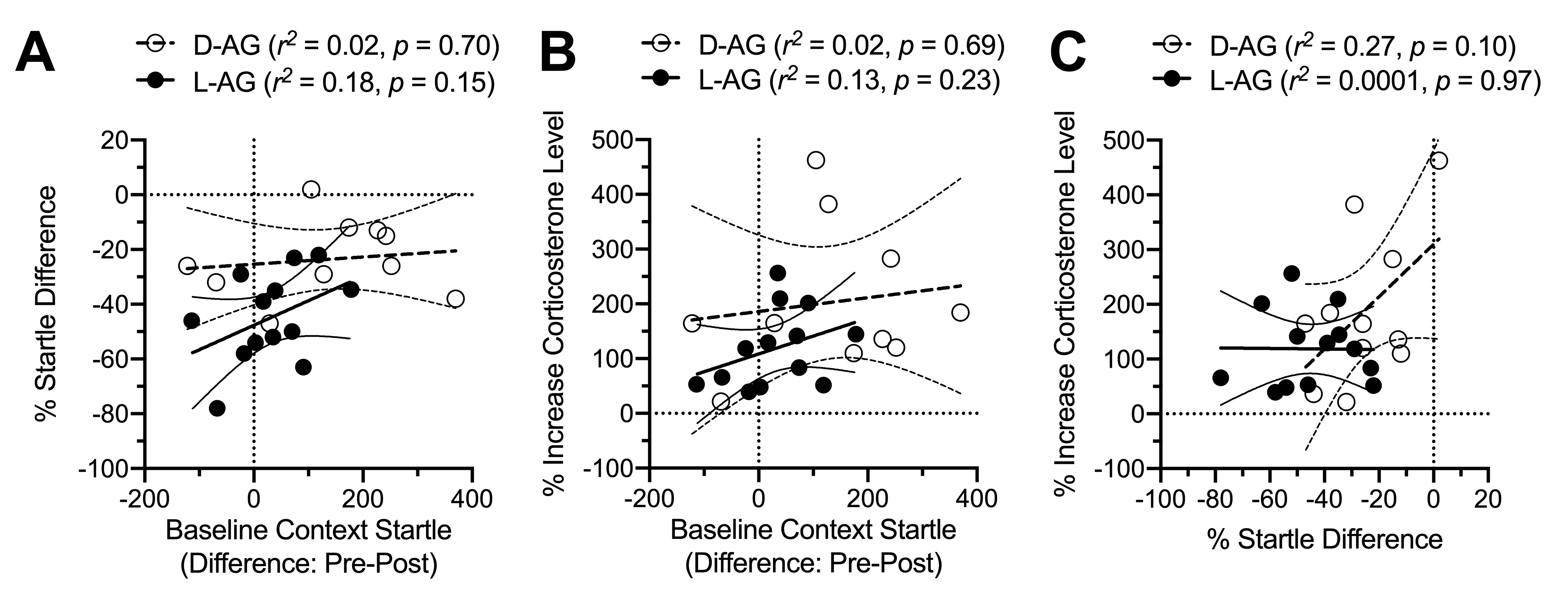
**

**Supplementary Figure S2**: **Correlation analyses of the individual baseline startle increase in the conditioning context, the effect of the safety CS on startle magnitude and the increase in plasma corticosterone level**

The impact of individual contextual fear, as represented by the difference of baseline startle between the pre-test and the post-test, on the individual startle attenuation by the safety CS (A) or the increase of CORT levels in the expression session (B) was assessed, as well as the impact of the increase of CORT levels on the safety CS effect (C). In D-AG treated control rats, context fear was neither correlated with the effect of the safety CS (A) nor the increase in CORT levels (B). The safety CS effect was correlated with the increase in CORT levels. In detail, diminished safety learning was associated with higher CORT levels (C). However, this correlation did only reach a trend level significance. In L-AG treated rats, very weak and non-significant correlations could be observed between the increase of baseline startle in the conditioning context and both, the effect of the safety CS on startle magnitude (A) and the increase in CORT levels (B). The effect of the safety CS on startle magnitude and the increase in CORT levels did not correlate (C). Open symbols represent individuals of the D-AG group, whereas filled symbols represent L-AG-treated rats. r2- and p-values in the diagrams indicate the results of a regression analysis.

**Supplementary Figure S3**

**
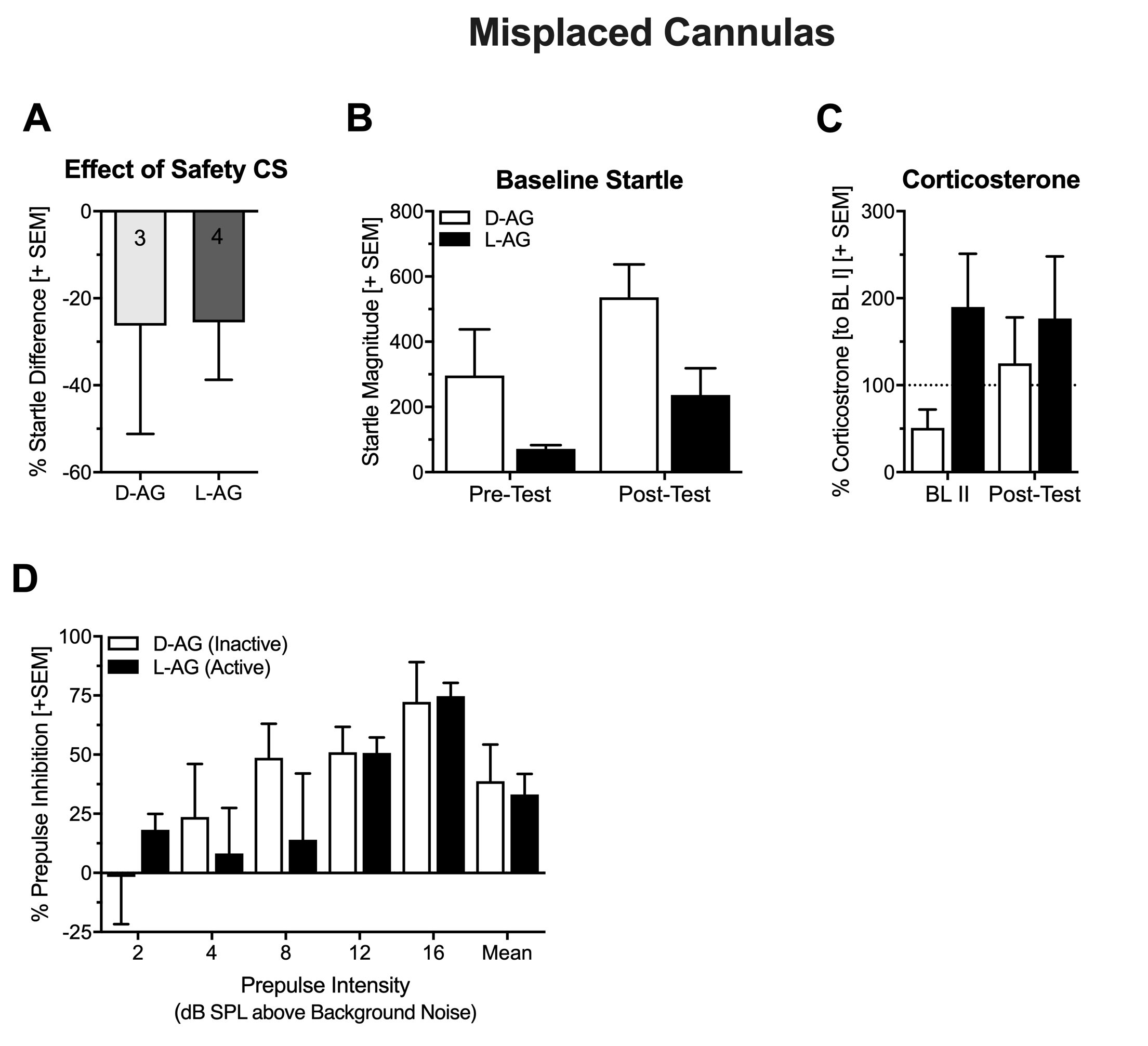
**

**Supplementary Figure S3**: **Misplaced cannulations aimed at the infralimbic cortex (IL)**In animals with misplaced infusion cannulas, chronic L-AG infusions did not affect the expression of conditioned safety (A), corticosterone (C) or prepulse inhibition (PPI) (D). Contextual baseline startle seemed to be reduced by L-AG infusions (B). The results indicate that our findings regarding conditioned safety, corticosterone and PPI were specific to IL manipulation. Data are represented as group averages + SEM.
